# Factors influencing the social perception of entrepreneurs in Spain: *A quantitative analysis from secondary data*

**DOI:** 10.1371/journal.pone.0296095

**Published:** 2023-12-20

**Authors:** Jose Luis Arroyo-Barrigüete, Carmen Escudero-Guirado, Beatriz Minguela-Rata

**Affiliations:** 1 Quantitative Methods Department, Universidad Pontificia Comillas, Madrid, Spain; 2 Business Management Department, Universidad Pontificia Comillas, Madrid, Spain; 3 Associate Professor of Business Administration Department, Complutense University of Madrid, Madrid, Spain; West Pomeranian University of Technology, POLAND

## Abstract

The essential role that entrepreneurs play in the economy, while acknowledged in the academic context, is not always recognized socially. In the specific case of Spain, the profession has even been called into question by public institutions, which is highly detrimental to both the social image of entrepreneurs and the country’s economy. Therefore, there is a need to identify factors that can improve this social image. This study aims to investigate one of these factors, specifically the effect of interest in economics. To do so, data from a large sample of 25,835 Spanish citizens from 2002–2022 were employed. The analysis was conducted using a combination of a neural network model and logistic regression. The conclusion of the study is that as interest in economics and business increases, the perception of entrepreneurs improves. Therefore, those individuals with less interest in economics tend to have a more negative view of this profession. This result opens up a potential avenue for enhancing the social image of entrepreneurs, as a higher interest in economics and business, which could be achieved through effective communication campaigns or basic education, leads to an improvement in perception.

## Introduction

Academic literature has highlighted the essential role played by the entrepreneur in a country’s economy and economic development. Among other aspects, entrepreneurs are a source of economic and social development, employment, innovation and competitiveness [1]. Previous research suggests that higher levels of entrepreneurship positively correlate with economic growth and productivity improvements [2]. Moreover, in recent years, some studies have also associated entrepreneurs with the achievement of sustainable development goals [3–5].

Different organizations and institutions have also highlighted the importance of promoting the figure of the entrepreneur and of enhancing the social recognition of the value they bring. For example, the EU [6] recognized that it remained essential that the role of entrepreneurs be perceived in a constructive way by all (political powers, institutions, the media, educational institutions, and society). The Global Entrepreneurship Monitor (GEM) considers culture, social values, and the degree of social recognition of entrepreneurial success as key drivers for the promotion of entrepreneurial vocations [7]. This is particularly important in the case of Spain, as its economic structure is composed almost entirely of small and medium-sized companies (99.81% of all companies) [8]. Entrepreneurs need resources and support from external stakeholders (individuals, companies, and government agencies, among others) both in business creation and survival [7,9–11]. Without such resources and support, success is unlikely to be achieved [12]. For external agents to provide resources and support, entrepreneurs and their companies need to be perceived as legitimate. [13, p. 574] defines legitimacy as desirability and appropriateness within a system of socially constructed norms, values, beliefs, and definitions. Regarding new ventures, legitimacy refers to the perception of how a firm’s relationship with other institutions aligns with societal norms and values [14]. However, these external agents have different perceptions when evaluating entrepreneurs based on their own norms, beliefs, rules, procedures, etc., making legitimacy a heavily audience-dependent construct [15].

According to [13], legitimacy can be explained differently (and sometimes contradictorily) from different theoretical perspectives, such as institutional theory, population ecology, or strategic approach. On the other hand, [16] identifies five perspectives for studying legitimacy: an institutional perspective, a corporate culture perspective, an ecological perspective, an impression management perspective, and a social movement perspective. According to this author, each audience may perceive and evaluate the legitimacy of entrepreneurs differently. This perception of an entrepreneur is considered one of the variables that contributes to creating a favorable ecosystem for business activity and entrepreneurship and, therefore, for the achievement of growth and economic development. Some authors have even cataloged the existence of a favorable climate for businesses and entrepreneurs as a public good and therefore desirable for the benefit of everyone [17].

Society’s appreciation for entrepreneurship involves valuing successful entrepreneurs, embracing the notion of failure, and fostering a positive entrepreneurial mindset, especially among educational institutions, financial backers, local communities, business entities, advisors, and the media. A key method to cultivate this mindset is to highlight success stories, making role models accessible and generating interest in entrepreneurship [18].

Attitudes and perceptions are not just personal; they are influenced by family, social values, and cultural traditions. It is important to advance the understanding of the variables that influence the development of a favorable entrepreneurial culture. Despite the increasing number of studies on entrepreneurial culture, there is still a need for broader research to identify, hypothesize, investigate, and confirm the relevant and appropriate variables that influence it [19].

Researchers have often overlooked and underestimated the societal viewpoint when forming social evaluations [20]. Since the figure of entrepreneur is perceived as a conflicted social archetype, the relevance of research related to social judgment is justified. Depending on the context, the entrepreneur is simultaneously associated with heroic and exploitative traits, while his or her critical economic role is recognized and associated with images that span from predators and exploiters to work machines, idea generators, and winners [11]. Some decisions made by entrepreneurs in the past have been criticized by the media, institutions, and other agents, while other external organizations/agents call for respect and the avoidance of personal disparagement. This perception of the possible demonization of the entrepreneurial role in recent years, specifically in Spain, may explain the gradual reduction in the number of experienced young entrepreneurs [21].

## Inclinations toward entrepreneurship and the social perception of entrepreneurs

There is now a rich body of research findings about entrepreneurship and the complexity inherent in its definition. Authors from different traditions and fields have agreed that entrepreneurship extends beyond the market economy, including a wide range of entities, from individuals to multinational corporations, as well as nonprofit organizations. However, no widely accepted definition of the term has been reached due to the differentiated perspectives and debates within the field of entrepreneurship research.

According to the seminal work of [22], two viewpoints on entrepreneurship can be highlighted: on the one hand, entrepreneurial situations are characterized by the involvement of individuals with unique characteristics (e.g., risk taking, locus of control, autonomy, perseverance, commitment, vision, creativity) and the presence of innovation, growth and uniqueness; on the other hand, key features of an entrepreneurial situation include value creation, for-profit characteristics, and a common owner-manager. Both viewpoints, as Gartner says, seem to reflect different parts of the same phenomenon [22].

[23] present a similar debate, with one view that considers the entrepreneur to be the person who creates and develops new businesses of any kind and a competing view that focuses on the entrepreneur-innovator as a relatively exceptional person.

[24] summarizes the different approaches and views on entrepreneurship, differentiating between entrepreneurship as an organizational context (based on criteria such as the size of the organization, self-employment, age, and whether it is owned by an individual—who is either self-employed or a new creator of the business—or a family -family ownership), entrepreneurship as defined by performance criteria (innovation or growth), and, finally, entrepreneurship as behavior (from the ability to recognize an opportunity and including exploitation and commercialization).

[25] synthesizes the key developments in entrepreneurship research, some of which have implications for the concept itself: a) the understanding of entrepreneurship as introducing new economic activities to the market, so that the restrictions of the association with independent, small, or new businesses are broken—this allowed for the expansion of the concept, incorporating new realities such as “corporate entrepreneurship” or “intrapreneuring”; b) entrepreneurship as a collective and social phenomenon beyond the individual; and c) the emergence of the "opportunity-based" entrepreneurship concept. Researchers such as [26] emphasized the identification and exploitation of opportunities as central to entrepreneurship. This view expanded the scope of entrepreneurship beyond just small business management and recognized it as a dynamic, opportunity-driven process.

The philosophical discussion of entrepreneurship is joined by another complementary approach focused on measurable characteristics commonly used to assess entrepreneurship. This perspective lies in the idea that not all concepts discussed in the entrepreneurship literature are easily measured and that pragmatic distinctions are needed between the realities that need to be measured [27]. [28] categorize entrepreneurs into three groups: intrapreneurs (working for others and driving a significant portion of incremental innovation in established large companies), managerial business owners (self-employed individuals representing most small businesses), and "Schumpeterian entrepreneurs" (innovation-driven individuals responsible for creative destruction).

In its attempt to build internationally comparable entrepreneurship statistics, the European Commission acknowledges the importance of having standard definitions for measurable concepts and conducts a nonexhaustive review since Richard Cantillon’s first recognized contribution to what we view today as entrepreneurship. From this review, various themes emerge, such as the risk-taking role of entrepreneurs, innovation and the creation of new things (processes, products, markets, or firms), entrepreneurial arbitrage, and the process of change, emergence, and creation. Despite variations, common definitions in the literature generally agree on the following points: entrepreneurship involves recognizing and acting on opportunities that generate value (economic, cultural, or social), and it implies the utilization of resources and capabilities through innovation, primarily related to identifying new products, processes, or markets [29].

This “practical” approach stems from the OECD’s “Entrepreneurship Indicators Programme” (EIP), according to which entrepreneurs are individuals (business owners) who aim to create value by discovering and capitalizing on new business opportunities. Entrepreneurial activity is human action in pursuit of the generation of value, and entrepreneurship is the overarching concept associated with these actions. While corporations and other enterprises can exhibit entrepreneurial behavior, only individuals who have both control and ownership of organizations are regarded as entrepreneurs.

The complexity of the concept of an entrepreneur, its inherent multidisciplinary nature, and the need to devise measures that serve as proxies have resulted in a wide-ranging understanding of what entrepreneurship is and who engages in it.

From the aforementioned perspective of entrepreneurship as a process that unfolds over time, entrepreneurial intentions would be the first step, as a necessary precursor to performing entrepreneurial behaviors. Following [30] widely used construct of entrepreneurial intention and according to Ajzen’s Theory of Planned Behavior [31], this intention depends on three motivational factors linked to perception: positive or negative personal evaluation about being an entrepreneur, social pressure from references who approve the decision to become an entrepreneur or not, and perception of the ease or difficulty of becoming an entrepreneur. This recognition of the relevance of perception, first of intention and later of behavior, directly aligns with the focus of our study, which is to deepen the understanding of the factors influencing opinions about entrepreneurs. According to [32], the importance of individual perceptions and culture in promoting entrepreneurial activity has been shown, but there has been no in-depth study of the aspects that contribute to improving these perceptions or culture.

Previously, researchers have examined how prior exposure and knowledge can influence individuals’ attitudes, making entrepreneurship a more desirable career choice and indicating a more favorable opinion.

To the best of our knowledge, there is a lack of academic studies that analyze the factors influencing opinions about entrepreneurs. This poses a significant challenge for this study, as it hinders the identification of relevant variables to include in the causal model. The objective of this research is to examine the impact of interest in economics on perceptions of the entrepreneur profession. However, this analysis must be conducted while controlling for other variables that may influence such perceptions, making it essential to identify them. Given this difficulty, an alternative approach has been proposed, focusing the literature review on entrepreneurial intention, which can be considered a proxy for perceptions, helping to identify the relevant variables. In other words, if we accept that a high intention to engage in entrepreneurship is likely a reflection of a positive view of the entrepreneur profession, variables that impact this entrepreneurial intention may also influence perceptions of entrepreneurs. Although this is a mere approximation, it allows us to approach the construction of the model in a structured manner.

Certainly, there is abundant work linking the terms "attitudes" and "entrepreneurs" and how individual factors (gender, income level, family tradition, etc.) and context (culture, institutional framework, access to finance, etc.) influence this relationship, with Ajzen’s theory [31] of planned behavior prevailing as an analytical framework [33] (see [34]) for a brief description of the theory). Starting with sociodemographic variables, features such as gender, household income and education can affect individual inclinations toward entrepreneurship [35,36]. The relationship between gender and entrepreneurship has been addressed in the literature from different perspectives. On the one hand, addressing the association of gender roles with different occupations and career expectations results in the categorization of jobs as predominantly feminine or masculine [37]. Based on stereotype threat theory, gendered stereotypes of entrepreneurship are firmly rooted in public beliefs and widely promoted in public discourse [38]. Stereotypes and societal norms discourage women from entrepreneurship as they anticipate negative stereotypes, shaping distinct career preferences and occupational choices for both genders based on perceived alignment [39]. On the other hand, the impact of gender is incorporated as a moderator in the perception of certain barriers to entrepreneurship [40,41]. In any case, there is no convergence in the influence of this variable when cross-cultural comparisons are addressed. Among the works that have addressed this issue [42], did not find incontestable conclusions about the cohesion and coherence of gender stereotypes regarding entrepreneurs even in the same society and asked for more research to explore the generalization of gender stereotypes about entrepreneurship to countries with contrasting norms on social roles for men and women. Therefore, it appears that gender is a variable that impacts entrepreneurial intention, but its directionality is not clear as it interacts with other variables in complex ways.

The relationship between education and entrepreneurship has been extensively addressed but without incontrovertible results [36]. On the one hand, it is assumed that a higher level of knowledge means that entrepreneurship is more highly valued as a desirable career option if these individuals have a business background [43–45]. However, other researchers suggest that formal education does not promote entrepreneurship [46]. In the middle positions, some authors suggest that education plays a moderating role in the relationship of other individual characteristics and variables to entrepreneurship [47].

The relationship between employment status and entrepreneurship is approached from two complementary perspectives: on the one hand, this relationship is part of the macro conditions that may favor (or hinder) entrepreneurship, referred to as entrepreneurial framework conditions [7,48], and on the other hand, it is one of the individual characteristics that may affect the perception, intention, and action of entrepreneurship [49]. Extant research has found differences between opportunity and necessity entrepreneurship. The former reflects start-up efforts to take advantage of a business opportunity because of the attractiveness of the activity itself [48,50], and the latter exists when there are "no better choices for work". Individuals with a more favorable socioeconomic situation tend toward opportunity entrepreneurship, while those in precarious employment situations tend toward necessity entrepreneurship. However, the interrelationship with other variables, both individual and contextual, nuances these tendencies [51].

The relationship between religion and entrepreneurship is intricate and mutually influential, so entrepreneurship studies cannot ignore important factors from the entrepreneur’s upbringing, such as their religious background [52,53]. The importance of studying religion in entrepreneurship research is growing into a nexus for discovering, pursuing, and capitalizing on opportunities to create future products and services driven by cultural and religious beliefs [54–56]. From a global perspective, religion influences the structure of values and social norms and can shape what is understood as a socially desirable (or undesirable) behavior [57,58]. According to [59], religious practices are expected to influence both individual and societal perspectives on entrepreneurial activities, given the inherent belief systems of religions and their relevance to societal objectives.

The literature presents contradictory results regarding the connections between religiosity and entrepreneurship [60]. Research has suggested that religious beliefs play a fundamental role, either positively or negatively, in shaping both the decision to engage in entrepreneurship and subsequent entrepreneurial endeavors [61–66]. This influence is reflected in the literature, either directly or as a moderator or mediator in the relationship with other variables. Either way, the argument to consider a link between religiosity and entrepreneurs is rooted in the idea that individuals often strive to integrate their religious and professional lives [67], leading them to avoid business practices that contradict their religious convictions [68,69]. Data analysis, which examined aggregated societal-level indicators of religiosity and GEM data, reveals correlations specifically with certain types of religion, particularly high-salience forms of Christianity [63]. According to [70], Christianity fosters an institutional framework that promotes self-employment endeavors by endorsing individualism and initiative and recognizing the significance of entrepreneurs. Conversely, other studies conclude that religion does not have a significant influence on the evaluation of entrepreneurship [67,71]. Nevertheless, despite the lack of consensus regarding the effect of this variable, it appears that religiosity can indeed have some impact and therefore should be included in the model.

Regarding socioeconomic levels, [72] (cited in [73]) pointed out that household income indirectly affects attitudes, intentions, and behaviors related to entrepreneurship. It seems that individuals coming from higher-income families tend to incline more toward entrepreneurship [74,75].

Related to this variable, we find political ideology. According to [76] and [77], political ideology is defined as a coherent and interconnected set of attitudes, moral values, and policies regarding the appropriate goals of society and how they should be achieved. Considering the values and beliefs associated with political ideology [76,78,79], the traditional way to classify this variable has been through a continuum, with the left/liberal wing on one end and the right/conservative wing on the other [79]. This way of characterizing the variable helps define the core values of individuals [76,79–81]. [79,81] indicate that the two key dimensions to differentiate liberal from conservative ideology are (1) attitudes toward inequality and (2) attitudes toward social change versus tradition. Individuals with a left/liberal ideological orientation are associated with concerns for civil rights and are more sensitive to the specific concerns of diversity, social change, human rights, and the environment and social issues in general [80]. This definition is complemented by the contributions of [79,81], which include individuals seeking to change the system, economic and social equality, solidarity, and control over markets. A right/conservative ideological orientation would include individuals who value individualism, property rights, free markets, and capitalism [76,79,81]. They highly value tradition, respect for authority, and order and have a high tolerance for economic inequality [76,77,81].

Considering the values associated with each type of ideology according to the literature, it is expected that individuals with a more right-leaning political ideology (individualism, property rights, free markets, capitalism, etc.) will be more likely to have a more positive social perception of entrepreneurs than those with a more liberal political orientation (economic and social equality, solidarity, controlled markets, human rights, etc.).

Finally, regarding the variable under study in this research, no evidence has been found in the literature on the potential relationship between interest in economics and business and its influence on the valuation of entrepreneurship. For the purposes of this paper, supported by cognitive theories of learning, a relationship between interest, knowledge and appreciation is proposed regarding entrepreneurs’ perceptions. According to [82], there is ample evidence that the presence of interest is foundational for both motivation and continued engagement with activities or content. The acquisition of knowledge transforms our perspective on the world. What may have once seemed monotonous and indistinguishable now becomes a wellspring of exhilaration and potential. Through our comprehension of the world, we find a sense of satisfaction and positive emotions, as we experience expertise and proficiency, leading to a genuine affinity for the subject [83]. Therefore, there is a link between interest, knowledge, and liking. As a result, and this is the hypothesis we aim to evaluate in this research, there may be a direct relationship between interest in economics and the valuation of entrepreneurs.

### Research objectives and hypothesis

The objective of this research is to explore the impact of interest in economics on social perceptions of entrepreneurship. Based on the literature described above, we propose the following research question:

RQ1: Does the level of interest in economics and business affect the social perception of entrepreneurs?

As previously explained, given the link between interest, knowledge, and preferences, our research hypothesis is expressed as follows:

H1: Individuals who express a higher level of interest in the field of economics and business will have a more positive perception of entrepreneurs.

To test this hypothesis, a quantitative research methodology was chosen using a nonexperimental design. Two types of causal models, neural networks and logistic regression, were employed on a sample of 25,835 individuals, following the procedure detailed in the next section.

## Material and methods

### Variables

The raw data were obtained from the "Surveys of Social Perception of Science and Technology in Spain," conducted biennially by the Department of Scientific Culture and Innovation of FECYT (Spanish Foundation for Science and Technology), under the Ministry of Science and Innovation of Spain. These data can be directly accessed from the foundation’s website [84]. Although this survey’s main objective is to deepen the understanding of the relationships between science, technology, and society, it provides a vast amount of information on many different topics. This is why it has been used in research on a wide variety of themes (see, for example [85–88]).

First, individuals over 18 years old were selected, except for 2012 and 2014, when the numerical data were not available and the information was provided in grouped intervals. In these two cases, it was not possible to exclude individuals younger than 18 years. However, the overall effect is minimal since in the years when the information was available, the number of records for individuals below 18 years old is very limited. Similarly, only individuals of Spanish nationality were included.

After this initial selection, a series of sociodemographic control variables based on the literature review developed in the previous section were incorporated. In cases where the variable was not available (the respondent answered "do not know" or "no answer"), the corresponding record was eliminated. The first variable is self-reported gender, coded as 1 for women and 0 for men. Political ideology has been included in the model as a categorical variable with three levels, and a transformation was necessary due to differences in the scale across the original surveys. In 2002 and 2004, ideology was measured on a scale from 0 (far left) to 10 (far right), while in the remaining years, it was measured from 1 (far left) to 10 (far right). Thus, for the former case, the categories considered were left (0–3), center (4–6), and right (7–10), and for the latter, they were left (1–4), center (5–6), and right (7–10). The level of education has also been incorporated as a categorical variable with three levels: elementary (education up to 14 years or less), high school (nonuniversity education), and university (university studies: bachelor’s, master’s, or Ph.D.). Employment status consists of five categories: employed (working for others or self-employed), retired, unemployed, unpaid work (housekeeper), and student. Regarding religiosity, the surveys from different years use slightly different categories, so a three-category scheme has been adopted: practicing Catholic, nonpracticing Catholic, and atheist or agnostic. Those who declared belief in another religion were excluded from the sample, as the number of records in this category was limited, making it impossible to carry out appropriate statistical analysis. The last considered control variable is household income, grouped into 5 categories. Since the measurement scale changed from year to year, it is difficult to find a completely homogeneous and valid categorization for all years. Therefore, an approximation has been employed. A 5-category scheme has been chosen: very low income (family income less than half the average family income in Spain), low (family income lower than the average family income in Spain), medium, high (family income higher than the average family income in Spain), and very high (family income more than double the average family income in Spain).

Regarding the variable under study in this research, interest in economics and business, the same scale used in the original surveys was employed. The data were obtained as a response to the question "Now I would like to know your level of interest in [economics and company affairs]: very low (1), low (2), moderate (3), high (4) or very high (5)." However, in 2022, a different scale was used, ranging from 0 (not interested at all) to 10 (very interested). For this reason, the data from that year were transformed to a scale of 1 to 5 according to the following criteria: 0, 1, 2 (1); 3, 4 (2); 5, 6 (3); 7, 8 (4); and 9, 10 (5).

Finally, the dependent variable was obtained as a response to the following question: "We’d like you to tell us how much you value each of the professions or activities I’m going to read out to you. For this, we will use a scale from 1 to 5, where 1 means that you value it very little and 5 means that you value it very much: Entrepreneurs."

### Sample

**Fig 1:**
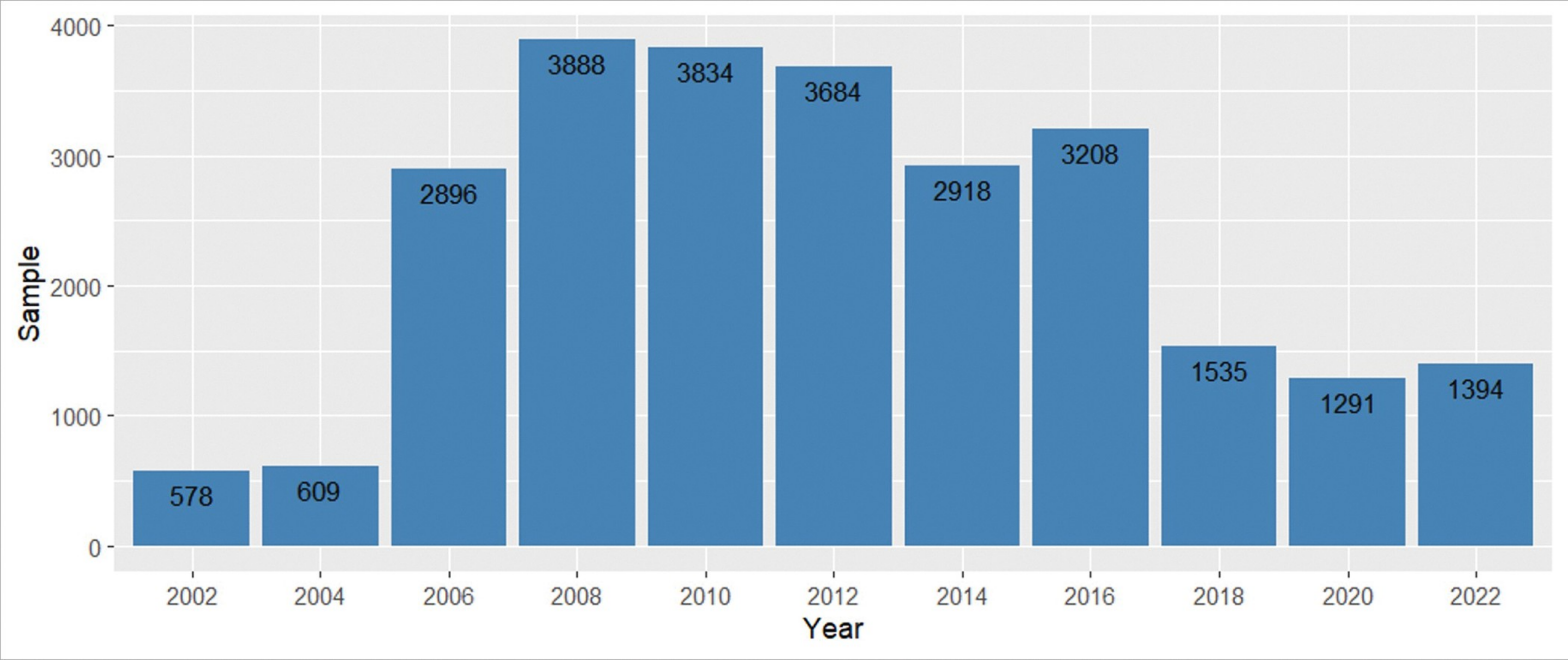
Characteristics of the sample, distinguishing each of the variables.

**Fig 2:**
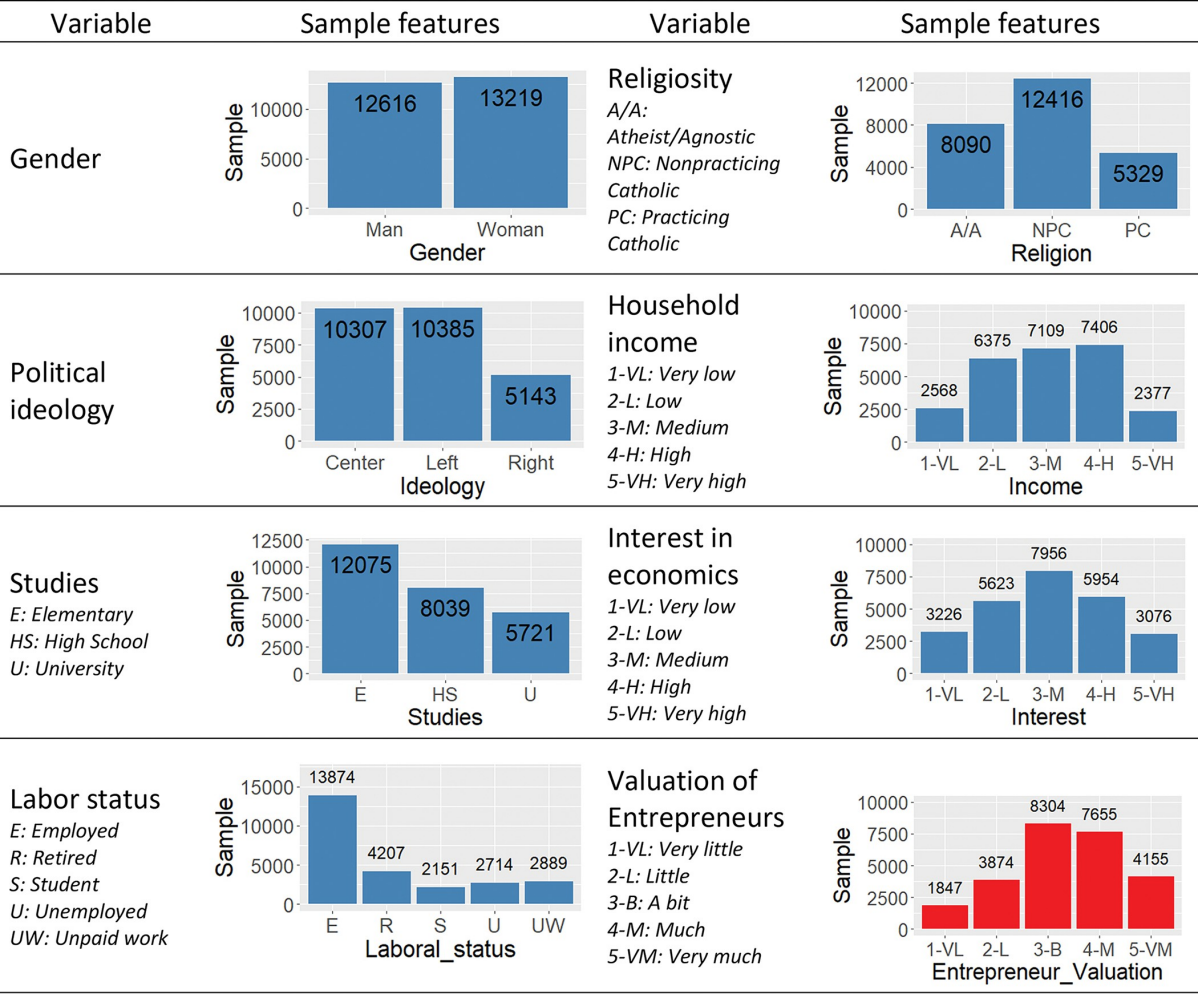
Distribution of the sample by year.

The considered sample includes all surveys on the social perception of science and technology in Spain conducted by FECYT in the period 2002–2022. Since these surveys are biennial, a total of 11 databases were used. After the cleaning and preparation process described in the previous section, the final sample consisted of 25,835 valid records (see Fig 1), with the distribution by year shown in Fig 2.

### Procedure

To develop the analysis, the basic functions included in the R programming environment were used [89] along with several packages: “caret” [90], “DescTools” [91], “dplyr” [92], “ggplot2” [93], “haven” [94], “NeuralSens” [95], “performance” [96], and “sjPlot” [97].

First, a crude analysis of the relationship between the dependent variable (perception of entrepreneurs) and the variable "interest in economics and company affairs" was conducted using a contingency table.

Next, a neural network (NN) model was fitted with the aforementioned variables. The variable of study, "interest in economics," was given a numerical treatment by standardizing the data. Specifically, two state-of-the-art algorithms were employed: the first one [95] allowed for a post hoc interpretation of the results of a neural network, and the second one [98] was capable of automatically identifying local effects in the input variables. Historically, the interpretability of NNs was relatively low, making their use for explanatory purposes unviable. However, recent developments in this field have opened the door to their utilization in explanatory models. Specifically, the algorithm proposed by [95] and implemented in the NeuralSens package allows for a truly simple post hoc interpretation of neural networks and is starting to be used in explanatory research in the social sciences [99]. In simple terms, the algorithm provides the slope of each independent variable, which would be equivalent to the beta coefficients in a linear regression model. However, instead of obtaining a single slope for each variable (a single beta), NN yields a slope (sensitivity) for each data point. This results in a sensitivity distribution for every predictor. In the absence of nonlinear relationships, this sensitivity distribution will be narrow (low standard deviation), and its mean will align precisely with the beta coefficient from a corresponding linear regression analysis. The advantage of using an NN model is that, without requiring an a priori functional specification, any nonlinear effect present in the data will be automatically identified by the network. Therefore, it is a valuable tool for validating the specifications of a conventional causal model. Additionally, the work by [98], based on alpha curves, provides a tool for detecting situations in which a certain independent variable that seemingly does not generate any effect (irrelevant variable) actually has an impact for a subgroup of data. In other words, it is capable of automatically identifying local effects of the input variables.

To fit the NN, we opted for the grid search method. This implied adjusting all the penalty combinations between 10^−4^ and 1 and selecting the NN with the highest accuracy. Since a certain tendency of overfitting was detected in the NN, we chose to use 1 perceptron in the hidden layer. Additionally, the 10-fold cross-validation procedure was applied. The optimal NN identified presents 1 perceptron in the hidden layer and a decay of 0.1. Once the optimal NN has been identified, the three sensitivity indicators proposed by [95] were calculated. The description of these metrics can be summarized as follows [95, p. 8]: mean sensitivity (MS) of the dependent variable with respect to the k-th independent variable (which, in the absence of nonlinear effects, would be equivalent to $\hat{\beta_{k}}$ in an OLS [ordinary least squares] model); sensitivity standard deviation (SSD) of the dependent variable with respect to the k-th independent variable (a metric that allows the identification of nonlinear effects when its value is high); and mean squared sensitivity (MSS) of the dependent variable with respect to the k-th independent variable (which is a measure of the relative importance of the k-th variable in the model). Finally, using the alpha curves algorithm developed by [98], the possible presence of local effects in the input variables was evaluated.

However, it is not possible to base the analysis solely on the NN. The reason is that NeuralSens is a brand-new technology that is not yet fully developed, and currently, it has a major shortcoming: it is not possible to develop the equivalent of a hypothesis test to evaluate the significance of the coefficients. This is the reason why, in its current state, NeuralSens must always be complemented with a conventional econometric model, in this paper a logit model, in which it is possible to test the significance of the variables.

In summary, we used a neural network (NN) with a post hoc analysis based on NeuralSens to identify any nonlinear relationship, whether we were aware of its existence or not. Subsequently, we used this knowledge to formulate a conventional econometric model, a logit, whose functional specification will be defined by the results of NeuralSens. It is the logit that allowed us to test the significance of the variables. In this particular study, this aspect is of great relevance because, due to the scarcity of quantitative studies on the topic, the functional specification of the model cannot be based solely on previous results. Therefore, a mixed approach, combining theory-driven and data-driven approaches, seems appropriate.

## Results

### Crude analysis

The crude analysis (Table 1) shows that the variables "interest in economics and company affairs" and "valuation of entrepreneurs" are not independent. In fact, if a numerical treatment is applied to both variables to calculate the correlation coefficient, it turns out to be positive and significant (r = 0.26, p value < 0.001). This is an initial indication that seemingly and in the absence of controlling for other factors, higher interest in economics implies a better valuation of entrepreneurs.

**Table 1:** Contingency table of the dependent variable (perception of entrepreneurs) and interest in economics and company affairs.

		How much do you value the profession of an entrepreneur?					
		Very little	A little	A bit	Much	Very much	Total
Interest in economics and company affairs	Very low	2.2%	2.6%	3.7%	2.4%	1.5%	**12.5%**
	Low	1.5%	4.8%	7.9%	5.2%	2.3%	**21.8%**
	Moderate	1.5%	4.0%	11.2%	9.8%	4.2%	**30.8%**
	High	1.1%	2.4%	6.4%	8.7%	4.4%	**23.0%**
	Very high	0.8%	1.1%	2.9%	3.5%	3.6%	**11.9%**
**Total**		**7.1%**	**15.0%**	**32.1%**	**29.6%**	**16.1%**	

### Causal model

**Fig 3:**
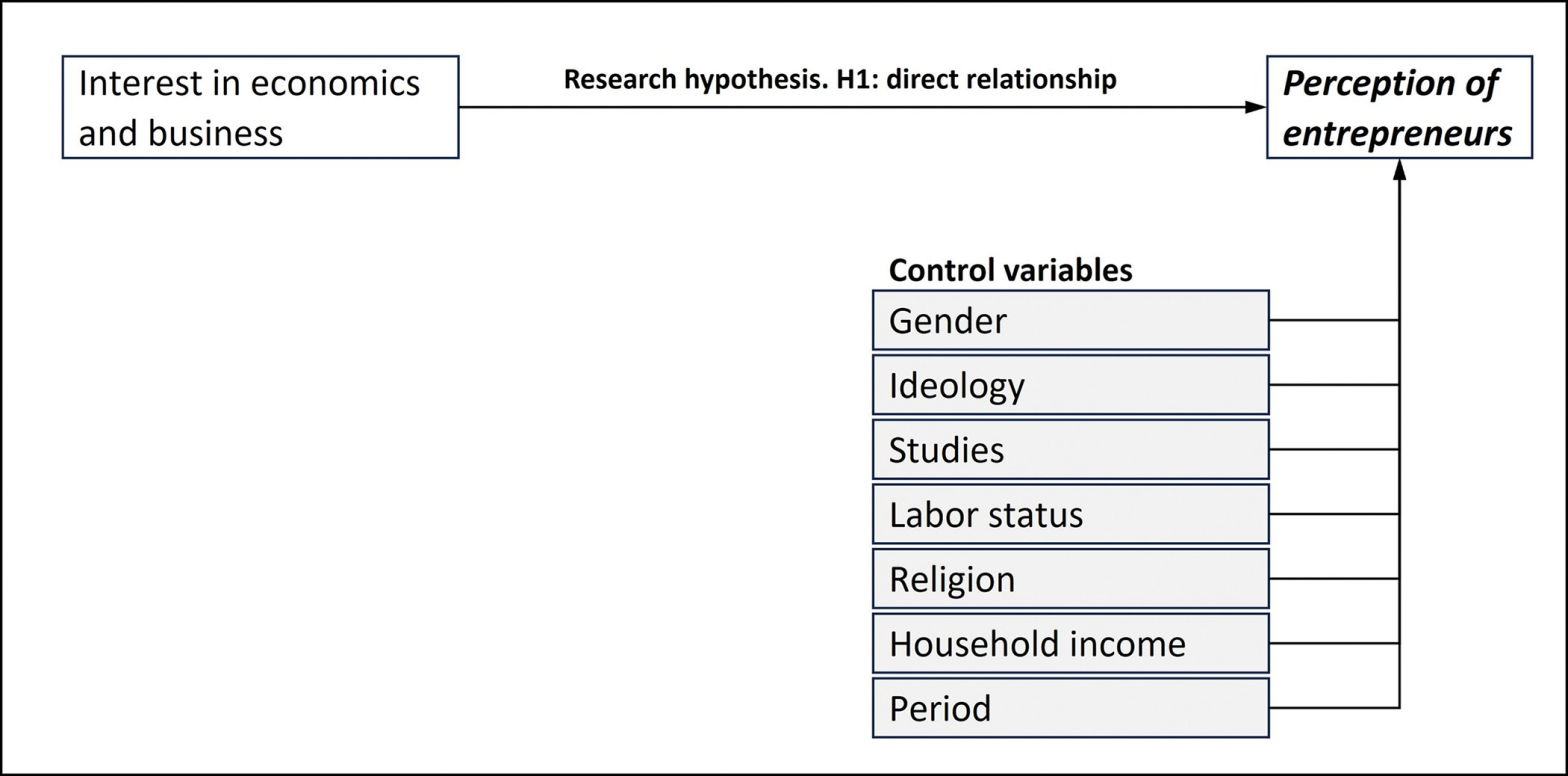
Theoretical diagram for the initial analytical model.

The initially proposed structure, taking into account both the variable under study (interest in economics) and the control variables (gender, ideology, studies, employment status, religion, household income, and period), is shown in Fig 3.

However, the NN model (Table 2) allows us to draw three conclusions regarding the effect of different variables. First, strong nonlinear effects are not observed, as the value of SSD is small for all variables. Nonetheless, in three variables ("Ideology," "Religion," and "Interest in economics"), the value is slightly higher, indicating the possible presence of a nonlinear effect. As previously explained, MS (mean sensitivity) is the mean value of the distribution of sensitivities and therefore an indicator of the intensity of the relationship between the dependent and independent variables. MMS (mean squared sensitivity) is an indicator of the importance of each variable. SSD (sensitivity standard deviation) is an indicator of the presence of nonlinear effects: high values point to the possible presence of some nonlinearity, as in this case for the three variables mentioned.

**Table 2:** NN model of the valuation of entrepreneurs. A value of 1 means a negative valuation (the value of this profession is “little or very little”), and 0 means neutral or good valuation (the value of this profession is “a bit, much, or very much”).

Predictors	MS	SSD	MMS
**Gender [male as base level]**			
Female	-0.03	0.01	0.03
**Ideology [center as base level]**			
Ideology [left]	0.06	0.03	0.07
Ideology [right]	-0.02	0.01	0.03
**Studies [elementary as base level]**			
Studies [high school]	0.00	0.00	0.00
Studies [university]	-0.01	0.01	0.01
**Labor_Status [employed as base level]**			
Labor_Status [retired]	0.02	0.01	0.02
Labor_Status [student]	-0.01	0.01	0.01
Labor_Status [unemployed]	0.03	0.01	0.03
Labor_Status [unpaid_work]	-0.02	0.01	0.02
**Religion [Atheist/Agnostic as base level]**			
Religion [Nonpracticing Catholic]	-0.04	0.02	0.05
Religion [Practicing Catholic]	-0.07	0.04	0.08
**Household income [high as base level]**			
Income [very low]	0.00	0.00	0.00
Income [low]	0.01	0.00	0.01
Income [medium]	0.01	0.01	0.01
Income [very high]	0.00	0.00	0.00
**Interest in economics and business**	-0.07	0.04	0.09
**Period [years 2002 to 2010 as base level]**			
Years 2012–2020	-0.03	0.02	0.03
Year 2022	-0.03	0.02	0.03

**Fig 4:**
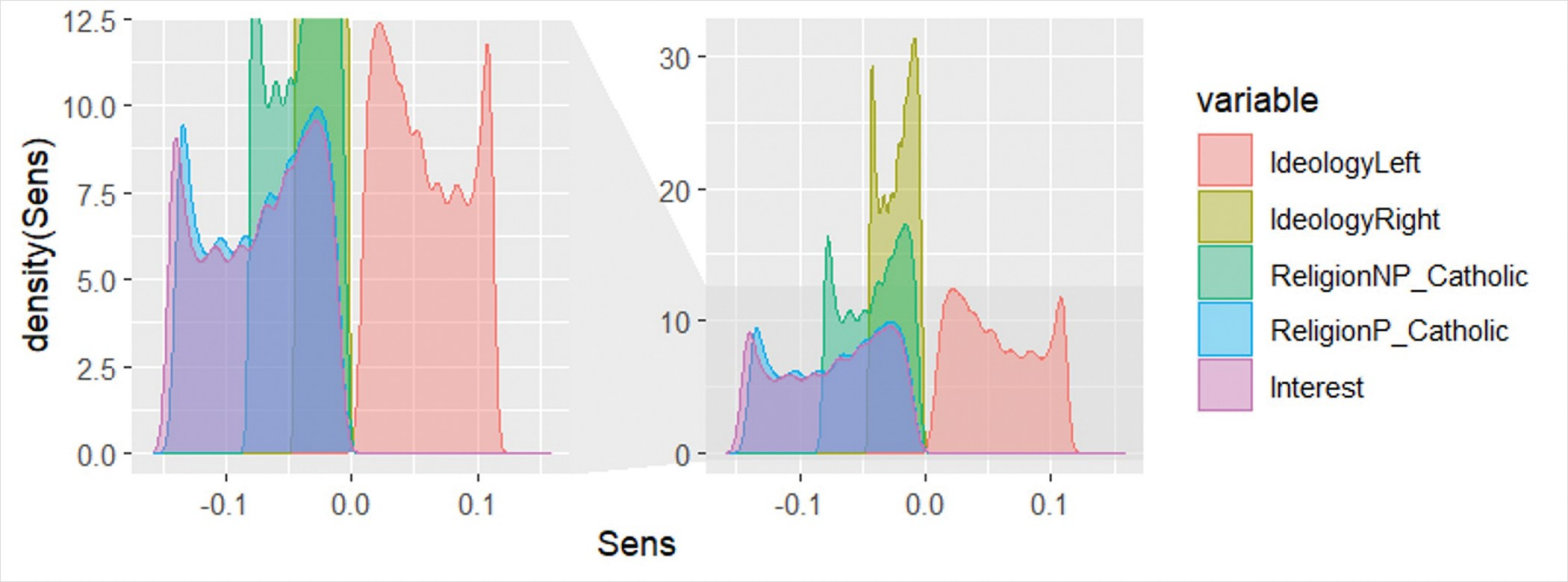
Sensitivity distribution in the NN for the most relevant variables.

**Fig 5:**
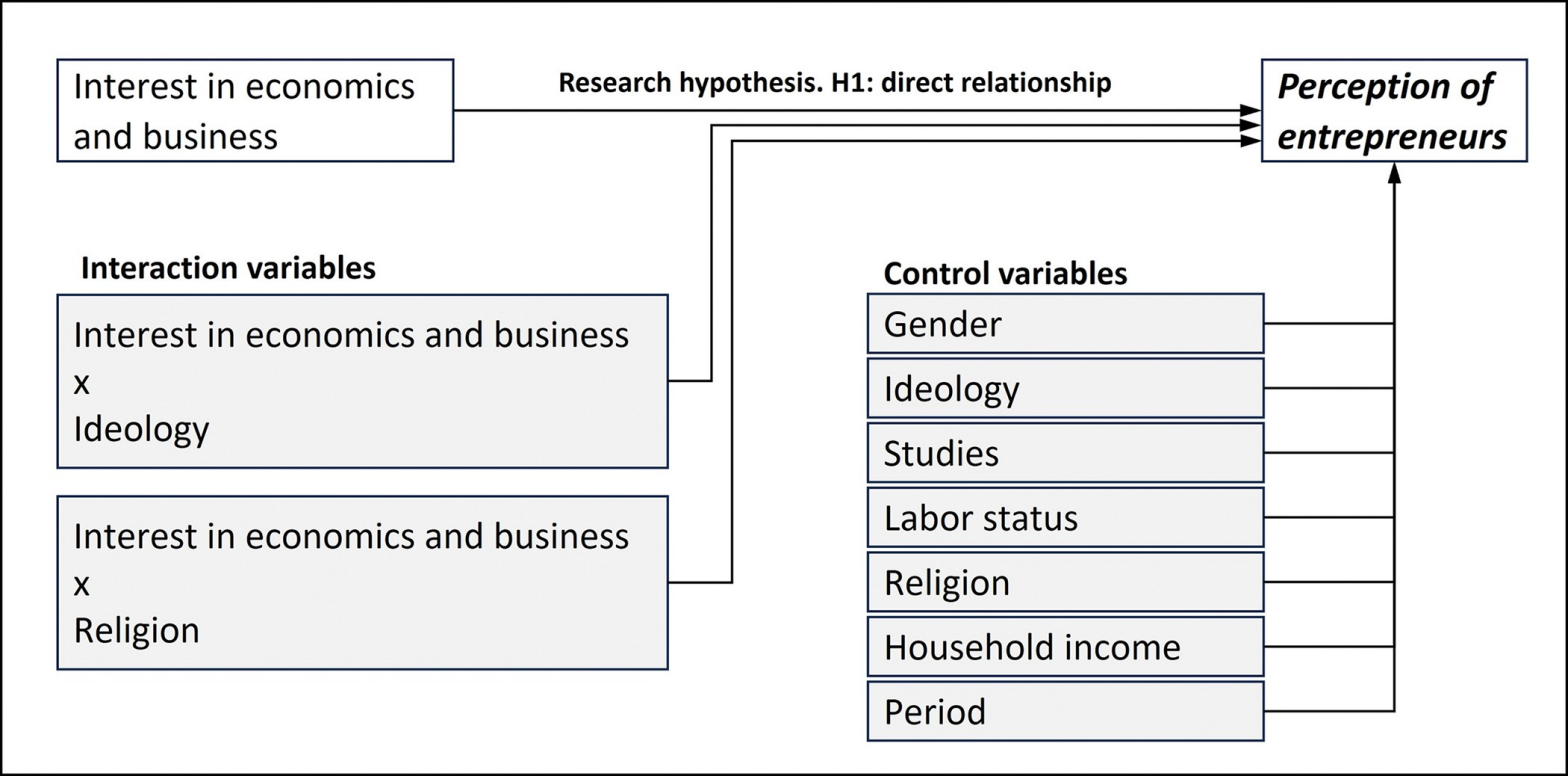
Theoretical diagram for the analytical model, considering the conclusions achieved after the post hoc analysis of the NN.

In fact, by analyzing the shape of the sensitivity distributions, it can be observed that they are multimodal (see Fig 4). This probably implies an interaction. Therefore, it seems reasonable to incorporate the interactions of these variables into the functional specification of the logistic regression model. This leads us to propose a modification to the functional specification of the logit model to be fitted in the next stage (see Fig 5).

Second, the three variables mentioned above appear to be particularly relevant, as their MS value is the highest among all the factors included in the model. Specifically, "Ideology [left], "Religion [Practicing Catholic]," and "Interest in economics" have a considerable effect on the negative valuation of entrepreneurs. In the case of the first variable, the effect is direct: individuals with a left-wing ideology have a higher probability of negatively valuing entrepreneurs. In the other two cases, the effect is negative: practicing Catholics have a lower probability of negatively valuing entrepreneurs, and as interest in economics increases, the probability of negatively valuing entrepreneurs decreases.

**Fig 6:**
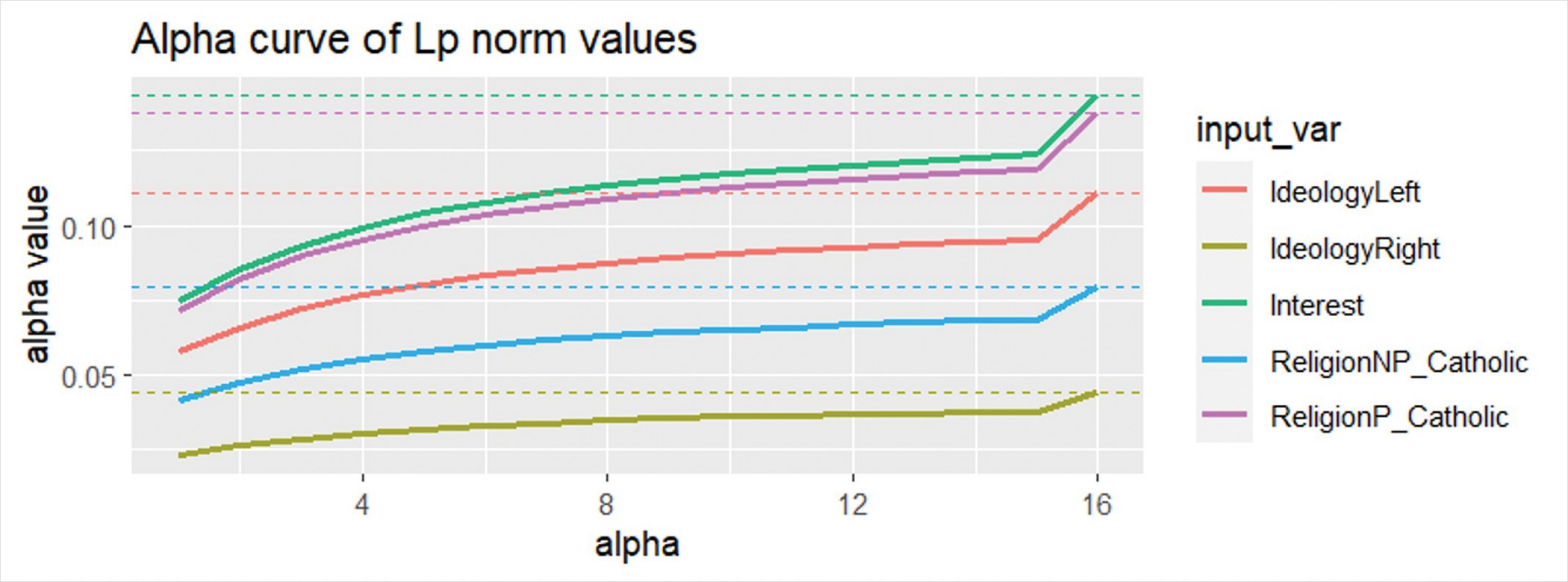
Alpha curves in the neural network for the most relevant variables.

Third, the analysis of alpha curves, a methodology proposed by [98], indicates that there are no local effects in the input variables. In other words, there is no variable whose effect, although not globally relevant, has a significant impact on a subset of data. Fig 6 displays the alpha curves for variables with a higher risk of local effects (more distorted alpha curves). This graph is interpreted as follows: the vertical axis represents the norm value for the sensitivity distribution of each variable, while the horizontal axis represents the considered norm, increasing from 1 (norm 1) to norm 15. The infinity norm is indicated at the value of 16 and represented by a dotted horizontal line. If there is a strong jump from norm 15 to the infinity norm or if the alpha curves intersect, it indicates the possible presence of local effects. As can be observed, this is not the case, allowing us to discard the presence of significant local effects in the variables.

Taking into consideration the information obtained from the NN, the logistic regression model shown in Table 3 has been fitted. Since it is a model with a binary dependent variable, it is not possible to directly compare the beta coefficients obtained in the logistic regression with the MS values in the NN (Table 2). However, as expected, it is observed that the signs are consistent in both cases, and the variables that are not significant in the logistic regression (p value > 0.05) also have MS and SSD values very close to 0 in the NN.

**Table 3:** Logistic regression model for the valuation of entrepreneurs. 1 means a negative valuation (the value of this profession is “little or very little”), and 0 means neutral or good valuation (the value of this profession is “a bit, much, or very much”).

Predictors	Log-Odds	Odds Ratios	P
**(Intercept)**	-1.15	0.32	**<0.001**
**Gender [male as base level]**			
Female	-0.17	0.85	**<0.001**
**Ideology [center as base level]**			
Ideology [left]	0.42	1.53	**<0.001**
Ideology [right]	-0.17	0.84	**<0.001**
**Studies [elementary as base level]**			
Studies [high school]	-0.01	0.99	0.769
Studies [university]	-0.07	0.94	0.140
**Labor_Status [employed as base level]**			
Labor_Status [retired]	0.13	1.14	**0.005**
Labor_Status [student]	-0.05	0.95	0.393
Labor_Status [unemployed]	0.19	1.20	**<0.001**
Labor_Status [unpaid_work]	-0.06	0.94	0.293
**Religion [Atheist/Agnostic as base level]**			
Religion [Nonpacticing Catholic]	-0.26	0.77	**<0.001**
Religion [Practicing Catholic]	-0.38	0.68	**<0.001**
**Household income [high as base level]**			
Income [very low]	0.04	1.04	0.487
Income [low]	0.10	1.10	**0.032**
Income [medium]	0.10	1.10	**0.02**
Income [very high]	-0.02	0.98	0.703
**Interest in economics and business**	-0.54	0.58	**<0.001**
**Period [years 2002 to 2010 as base level]**			
Years 2012–2020	-0.19	0.82	**<0.001**
Year 2022	-0.22	0.80	**0.003**
**Interaction Interest—Ideology**			
Interest * Ideology [left]	0.09	1.10	**0.009**
Interest * Ideology [right]	-0.06	0.94	0.192
**Interaction Interest—Religion**			
Interest * Religion [Non-Practicing Catholic]	0.07	1.07	0.063
Interest * Religion [Practicing Catholic]	0.19	1.21	**<0.001**
R^2^ Nagelkerke	0.08		

**Fig 7:**
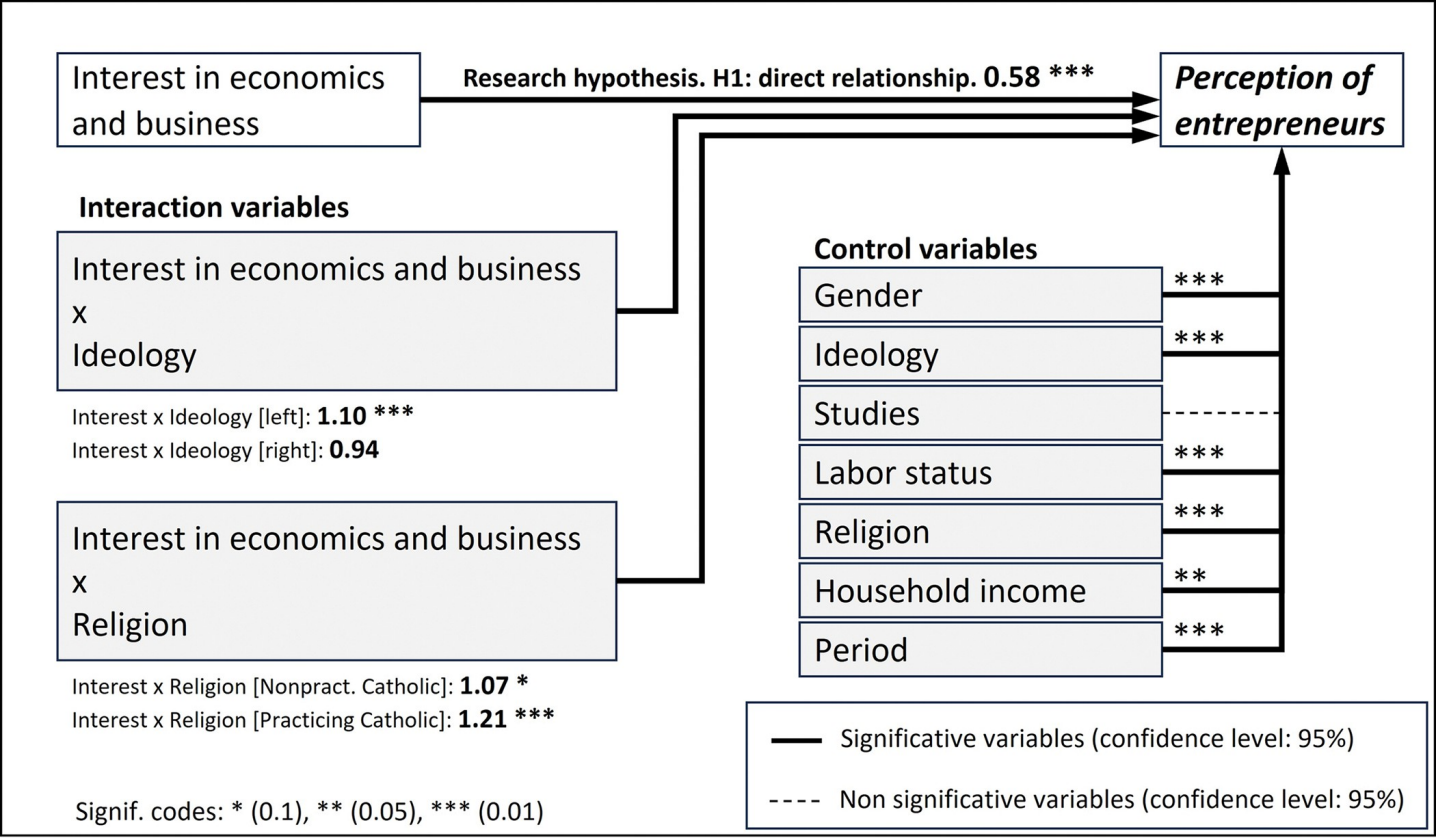
Theoretical diagram for the analytical model, including the results obtained in the logit model.

The negative sign of the coefficient for the variable of study, interest in economics and business, as well as the fact that it is significant, confirms the research hypothesis: individuals who have a higher level of interest in the field of economics and business have a more positive perception of entrepreneurs (the dependent variable takes the value of 1 when the perception is negative). Even in those cases where the interaction variable has the opposite sign (positive), the combined effect is not fully compensated, and the conclusion remains the same. Fig 7 schematizes the results obtained.

## Discussion

This study aimed to explore the impact of interest in economics and business on social perceptions of entrepreneurship while controlling for a range of sociodemographic and individual variables. The value of this study lies in its attempt to fill a research gap regarding the factors that influence opinions about entrepreneurs. The results of the study are compelling and provide a basis for further investigation. Our analysis, employing both a neural network model interpreted with NeuralSens post hoc analysis and logistic regression, allows us to draw several conclusions.

In relation to the control variables, it is observed that men are more likely to have a negative view of entrepreneurs. As we noted in the literature review, gender is a variable that impacts entrepreneurial intention, but its directionality is not clear. This study concludes that, regarding this profession, women seem to hold higher opinions than men. On the other hand, unemployed or retired individuals also show a greater propensity to have a negative image of entrepreneurs than employed individuals.

Additionally, there is a certain trend that as income level increases, perception toward entrepreneurs improves. However, this trend is not entirely clear since, in some of the categories included in this variable, the corresponding coefficient does not turn out to be significant. Previous studies have indicated that individuals coming from higher-income families tend to lean more toward entrepreneurship [74,75]. Our results, although not fully conclusive, do seem to be aligned with this, as the view toward entrepreneurs appears to improve with an increase in income level.

Two other control variables are particularly relevant, as they generate a considerable impact: "Ideology" and "Religion". As expected, our findings revealed that individuals with a left-wing ideology were more likely to hold negative views about entrepreneurs. Conversely, Catholics, both practicing and nonpracticing, were less likely to harbor negative perceptions about entrepreneurs than atheists and agnostics. As we previously mentioned, according to [70], Christianity fosters a more positive view of entrepreneurs. Our results seem to confirm this, contrary to what was suggested by [67,71], who concluded that religion does not have a significant influence on the evaluation of entrepreneurship.

The variable of interest in this research, "Interest in economics and business," turned out to have the greatest impact. Individuals with a high interest in economics were less likely to harbor negative perceptions about entrepreneurs than those disinterested in economics.

The existence of an interaction effect between the "Interest in economics and business" variable and the “Ideology” and “Religion” variables is very interesting. Regarding the first interaction, it is observed that among practicing Catholics, the effect of the "Interest in Economics and business" variable is less than in other groups. That is, as “Interest in Economics and business” increases, the perception of entrepreneurs improves, but to a lesser extent than for the rest of the groups (Nonpracticing Catholics and Atheist/Agnostics). The same situation applies to left-leaning individuals: as the “Interest in economics and business” increases, the perception of entrepreneurs improves, but to a lesser extent than for center or right-leaning individuals.

It is important to highlight that these results do not indicate strong nonlinear effects for any variables beyond the mentioned interactions. There is also no evidence of local effects in the input variables, suggesting a uniform impact across the dataset. This has been verified through the analysis of sensitivity distributions in the neural network and with the analysis of the alpha curves, both of which are recently developed state-of-the-art algorithms [95,98]. This finding reinforces the robustness of our model and the relevance of the identified variables across diverse demographic and individual contexts.

## Conclusions

The objective of this research was to examine the impact of interest in economics and business on perceptions of the entrepreneur profession. The research hypothesis proposed that individuals who express a greater interest in economics and business will have a more positive perception of entrepreneurs. It is observed that indeed, a higher interest in economics leads to a more positive perception of entrepreneurs, thus confirming the research hypothesis. This result is consistent with expectations derived from previous studies. According to [82], there is ample evidence that the presence of interest is foundational for both motivation and continued engagement with activities or content. Consequently, the feeling of mastery manifests itself in positive emotions and a genuine affinity for the subject matter we have mastered [100]. Thus, there is a link between interest, knowledge, and preference. This is precisely what we have been able to verify in the present research, confirming that there exists a direct relationship between interest in economics and the valuation of entrepreneurs.

In summary, this study primarily reveals that individuals with a heightened interest in economics and business generally view entrepreneurs more favorably. While several sociodemographic factors, such as ideology, religiosity, gender, employment status, and income level, influence the perceptions of entrepreneurs, most of these are not readily changeable. However, one’s interest in economics is malleable, suggesting potential for initiatives that encourage this interest and thereby improving such perceptions. From a methodological perspective, this study also makes a significant contribution, as it employs a novel post hoc analysis for neural networks, NeuralSens [95], and is also the first study to use alpha curve analysis [98] for the detection of local effects.

This finding has clear implications. Certain sociodemographic variables, such as ideology, religiosity, gender, employment status, or income level, have a significant impact on the perception of entrepreneurs. However, none of these variables are easily modifiable. In contrast, the level of interest in economics can be modified, and it is precisely this variable that has the greatest impact on the perception of entrepreneurs. This opens the door to considering initiatives aimed at promoting such interest.

One potential initiative, beyond the proliferation of university studies related to economics, could be to promote the inclusion of economics subjects in the early stages of compulsory education. In this regard, in 2003, the Círculo de Empresarios published a research study titled "El empresario y la economía de mercado. Breve recorrido por los textos de Historia, Geografía y Economía utilizados en los centros de Enseñanza Media" ("The Entrepreneur and the Market Economy: A Brief Overview of History, Geography, and Economics Textbooks Used in Secondary Education Centers"). The main conclusion of this work was that there was perceived hostility toward entrepreneurship. These textbooks presented a negative image of entrepreneurs contrary to their positive contribution to the economy. Subsequently [101], identified a systemic absence of the figure of the entrepreneur and their contribution to society in the analyzed textbooks. Addressing these issues appears to be an appropriate measure to improve the perception of entrepreneurs.

The second way to improve the public image of entrepreneurs is closely related to campaigns and media communication. In the case of Spain, and especially in recent times, business associations have called for calm in response to negative messages spread in the media about their activities. The clearest example is found in the statement issued by CEOE (the main association of companies in Spain) on this matter (see [102]), stating that they "deeply regret the unfair strategy of discrediting and disparaging Spanish entrepreneurs." While such statements may be timely, the communications of business organizations should not only focus on reducing negative messages about entrepreneurs but also aim to promote greater interest in economic matters. As we have seen in this research, interest is a powerful tool to improve the social perception of entrepreneurs.

To verify the conclusions reached in this work, a new research approach is proposed as a future line of research: an experimental design. That is, a new design should evaluate to what extent the presence and intensity of a stimulus capable of generating a greater interest in economics and business improves the perception of entrepreneurs.
